# Pressor response to angiotensin II is enhanced in aged mice and associated with inflammation, vasoconstriction and oxidative stress

**DOI:** 10.18632/aging.101255

**Published:** 2017-06-28

**Authors:** Quynh Nhu Dinh, Grant R. Drummond, Barbara K. Kemp-Harper, Henry Diep, T. Michael De Silva, Hyun Ah Kim, Antony Vinh, Avril A.B. Robertson, Matthew A. Cooper, Ashley Mansell, Sophocles Chrissobolis, Christopher G. Sobey

**Affiliations:** ^1^ Cardiovascular Disease Program, Biomedicine Discovery Institute, Department of Pharmacology, Monash University, Victoria, Australia; ^2^ Department of Surgery, Monash Medical Centre, Southern Clinical School, Monash University, Victoria, Australia; ^3^ The Institute for Molecular Bioscience, The University of Queensland, Queensland, Australia; ^4^ Current affiliation: Department of Physiology, Anatomy and Microbiology, School of Life Sciences, La Trobe University, Victoria, Australia; ^5^ Current affiliation: Department of Pharmaceutical and Biomedical Sciences, Raabe College of Pharmacy, Ohio Northern University, Ohio, USA; ^6^ Hudson Institute of Medical Research, Victoria, Australia

**Keywords:** aging, hypertension, angiotensin II, inflammation, oxidative stress, vasoconstriction

## Abstract

Aging is commonly associated with chronic low-grade inflammation and hypertension but it is unknown whether a cause-effect relationship exists between them. We compared the sensitivity of young adult (8-12 w) and aged (23-31 mo) male C57Bl6J mice to develop hypertension in response to a slow-pressor dose of angiotensin II (Ang II; 0.28 mg/kg/d; 28 d). In young mice, the pressor response to Ang II was gradual and increased to 142±8 mmHg over 28 d. However, in aged mice, Ang II promptly increased SBP and reached 155±12 mmHg by 28 d. Aging increased renal but not brain expression of Ang II receptors (*At1ar* and *At2r*) and elevated AT1R:AT2R expression ratio in mesenteric artery. Maximal contractile responses of mesenteric arteries to Ang II were enhanced in aged mice and were not affected by L-NAME, indomethacin or tempol. Mesenteric arteries and thoracic aortae from aged mice exhibited higher Nox2-dependent superoxide production. Despite having higher renal expression of *Nlrp3, Casp-1* and *Il-1β*, Ang II-induced hypertension (SBP: 139±7 mmHg) was unaffected by co-infusion of the NLRP3 inflammasome inhibitor, MCC950 (10 mg/kg/d; SBP: 145±10 mmHg). Thus, increased vascular AT1R:AT2R expression, rather than NLRP3 inflammasome activation, may contribute to enhanced responses to Ang II in aging.

## INTRODUCTION

There is a high global prevalence of hypertension, especially in advanced age, such that more than half of the elderly population (>65 years) have diagnosed hypertension [1]. The renin-angiotensin-system is an important mechanism contributing to blood pressure regulation, and there is evidence that its activity is upregulated in aging. For example, despite no change in basal blood pressure [2], aged mice are reported to exhibit an enhanced pressor response to angiotensin II (Ang II) [3] and increased renal expression of the Ang II type 1 (AT1) receptor – the main target receptor of Ang II for promoting hypertension [3].

Inflammation is associated with hypertension [4, 5], although it remains unclear as to whether a cause-effect relationship exists. Inflammatory responses may be initiated by inflammasome complexes, which activate caspase-1 to promote the maturation of the interleukin (IL)-1 family cytokines IL-1β and IL-18, which ultimately stimulate the release of pro-inflammatory cytokines such as IL-6 and interferon (IFN)-γ. NOD-like receptors (NLR) are a type of pattern recognition receptor (PRR), and make up part of an inflammasome complex, the best characterized being the NOD-like receptor family pyrin domain-containing protein (NLRP) 3 inflammasome, which is composed of pro-caspase-1, the PRR, NLRP3 and the adaptor protein, ASC [6]. Our laboratory has reported that treatment with MCC950, a selective NLRP3 inflammasome inhibitor, can reverse deoxycorticosterone acetate (DOCA) salt-induced hypertension in mice [7], implicating a role for inflammasomes in the development of at least one form of hypertension. It is noteworthy that compared with young adult rats, greater renal expression of NLRP3 and caspase-1 is reported to occur in old rats [8] and so it is plausible that inflammation-driven hypertension is augmented in old age.

Here, our first aim was to test whether aged mice treated with a ‘slow-pressor’ low dose of Ang II – which causes a gradual increase in blood pressure in young adult mice [9] – exhibit enhanced pressor responses. The second aim was to test whether aged mice have greater expression of renin-angiotensin system and NLRP3 inflammasome components in association with exaggerated end-organ inflammation, vascular oxidative stress and vasoconstriction. Thirdly, we tested the effect of MCC950, an NLRP3 inhibitor, on Ang II-induced hypertension in aged mice.

## RESULTS

### Effect of Ang II on blood pressure in young and old mice

**Figure 1:**
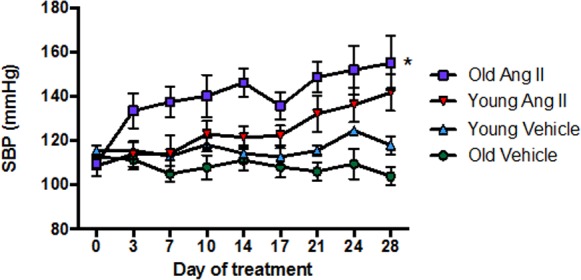
Effect of aging on the pressor response to a slow-pressor dose of angiotensin II (Ang II) (n=8-9) All data are mean ± S.E.M. *P<0.05 vs young Ang II-infused, one-way repeated measures ANOVA with Tukey’s post-hoc test.

In young mice, there was little or no effect of Ang II infusion on systolic blood pressure (SBP) for more than 7 d, after which SBP gradually increased by ~30 mmHg over the remaining 21 d of treatment (Fig. 1). By contrast, in aged mice SBP promptly increased by >20 mmHg within 3 d of commencement of Ang II infusion, and it had increased by >40 mmHg by 28 d (P<0.05 vs young). Infusion of saline (vehicle) had no effect on SBP in either young or aged mice over 28 d (Fig. 1).

### Gene expression of components of the renin-angiotensin system in young and old mice

**Figure 2:**
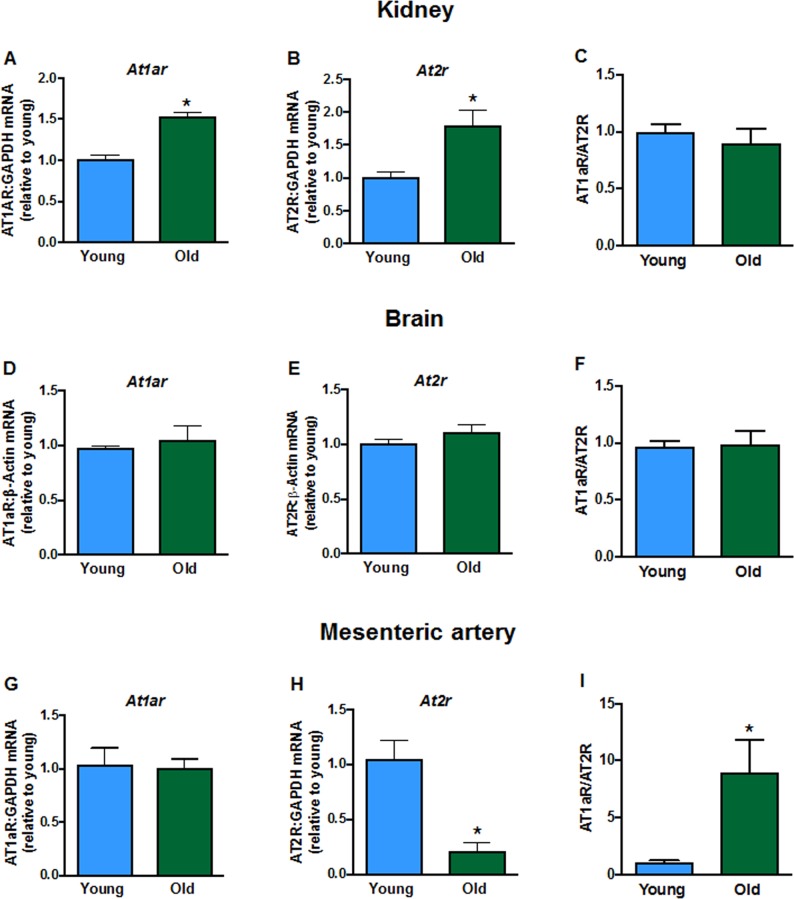
Renal mRNA expression of angiotensin receptors **A**: type 1a (*At1ar)* and **B**: type 2 (*At2r)* in young and aged mice (n=7-8). Brain mRNA expression of **D**: *At1ar* and **E**: *At2r* in young and aged mice (n=7-8). Mesenteric artery mRNA expression of **G**: *At1ar* and **H**: *At2r* in young and aged mice (n=6-7). Effect of aging on *At1ar:At2r* ratio in **C**: kidney, **F**: brain and **I**: mesenteric artery (n=6-8). All data are mean ± S.E.M. *P<0.05 vs young, Student’s unpaired t-test.

Aging elevated mRNA expression of the AT1a receptor (*At1ar*) in kidneys (Fig. 2A) of aged mice but had no effect in brain (Fig. 2D) or mesenteric arteries (Fig. 2G). AT2 receptor (*At2r*) mRNA expression was increased in kidneys (Fig. 2B), not different in brains (Fig. 2E), and markedly reduced in mesenteric arteries (Fig. 2H) from aged mice compared to young mice. As a result, aging markedly increased the AT1aR/AT2R expression ratio in mesenteric arteries (Fig. 2I) but the ratio was not changed in kidney (Fig. 2C) or brain (Fig. 2F). There were also lower levels of *Ace* (Supplemental Fig. 1A) and *Renin* (Supplemental Fig. 1B) expression in kidneys from aged mice. Aging did not affect expression of the Mas1 receptor (*Mas1*, Supplemental Fig. 1C) or *Angiotensinogen* (Supplemental Fig. 1D).

### Effect of aging on vascular tone and superoxide production in arteries from young and old mice

**Figure 3:**
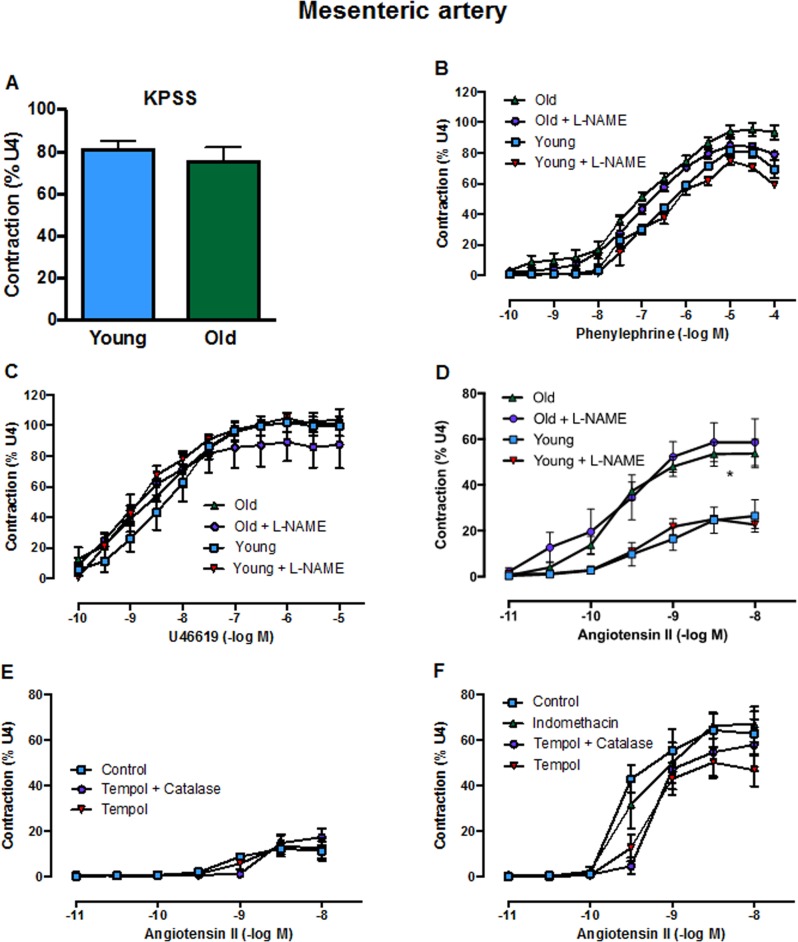
Contractile responses of the mesenteric artery to **A**: KPSS, **B**: phenylephrine **C**: U46619 (U4) and **D**: angiotensin II (Ang II) in the absence or presence of L-NAME in young and old mice (n=3-6). Contractile responses to Ang II in the absence or presence of tempol, tempol + catalase or indomethacin in **E**: young mice and **F**: aged mice (n=3). All data are mean ± S.E.M. *P<0.05 vs young, two-way ANOVA with Tukey’s post-hoc test.

**Figure 4:**
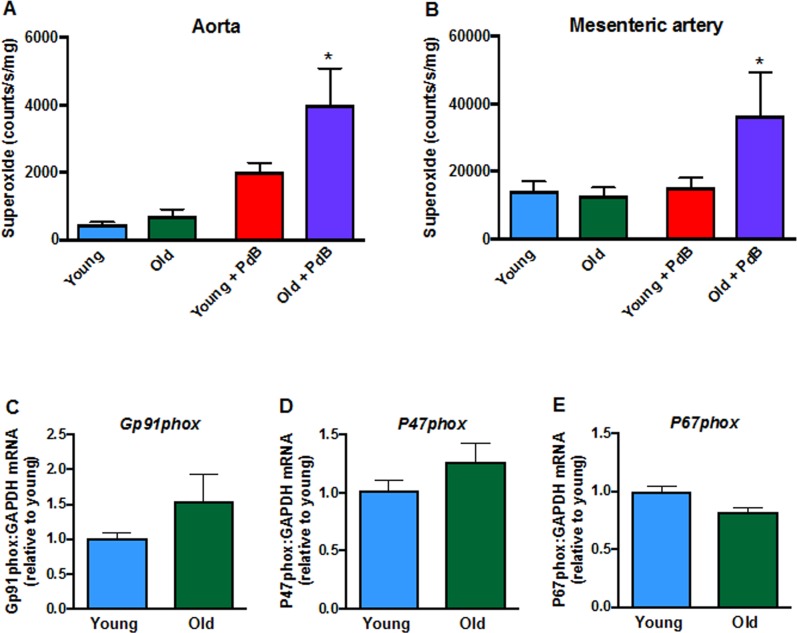
Superoxide levels in **A**: aorta (n=9-10) and **B**: mesenteric arteries (n=6) measured in the absence or presence of phorbol-dibutyrate (PdB). Mesenteric artery mRNA expression of NOX2 oxidase subunits **C**: NOX2 (*Gp91phox),* **D**: *P47phox* and **E**: *P67phox* in young and aged mice (n=6-7). All data are mean ± S.E.M. *P<0.05 vs young + PdB, one-way ANOVA with Bonferroni’s post-hoc test.

Contractile responses of mesenteric arteries to high potassium solution (KPSS) (Fig. 3A), phenylephrine (Fig. 3B) and U46619 (Fig. 3C) did not differ between young and aged mice. However, there was a markedly greater contractile response to Ang II in arteries from aged mice (Fig. 3D). Acute pre-treatment with L-NAME had no effect on contractile responses to either phenylephrine, U46619 or Ang II in young or old mice. Moreover, contractile responses to Ang II were also not affected by indomethacin (aged mice only), tempol or tempol + catalase in either young or aged mice (Fig. 3E, F). Basal levels of superoxide did not differ between young and aged mice in either aorta (Fig. 4A) or mesenteric arteries (Fig. 4B). Stimulation of NOX2 oxidase activity using PdB resulted in significantly higher levels of superoxide production in both the aorta and mesenteric arteries of aged mice compared with young mice (Fig. 4A, B). The increase in capacity for NOX2-dependent superoxide production in the mesenteric arteries of aged mice was not associated with any change in expression of several genes associated with NOX2 oxidase activity (*Gp91phox*, *P47phox*, *P67phox)* (Fig. 4C-E).

### Gene expression of pro-inflammatory markers in young and old mice

**Figure 5:**
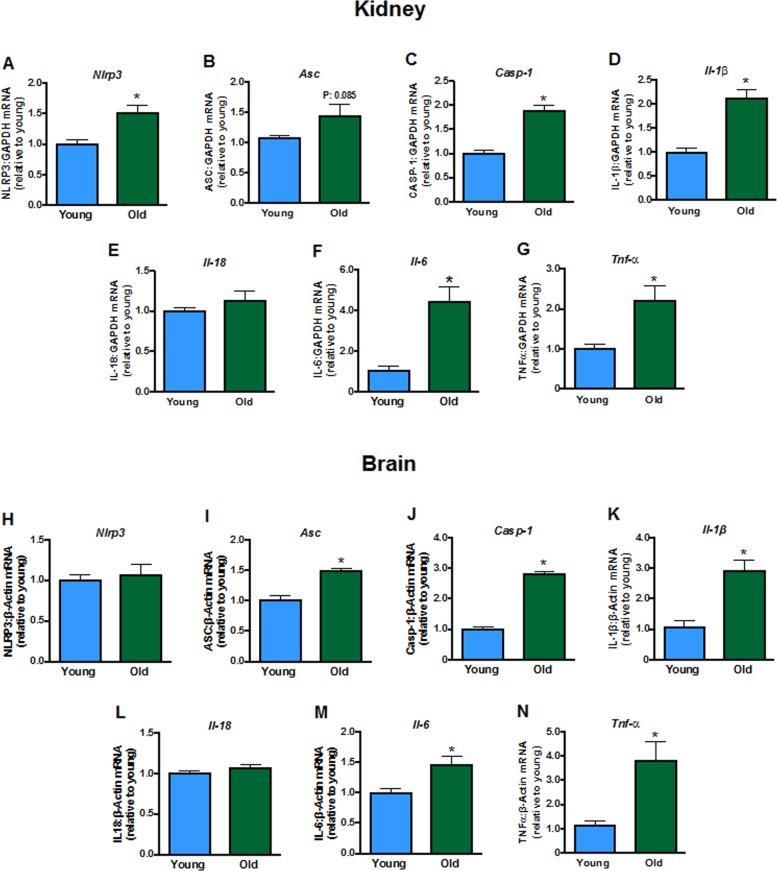
Renal mRNA expression of inflammasome components **A**: NLRP3 *(Nlpr3)*, **B**: *Asc*, **C**: caspase-1 *(Casp-1),* interleukins **D**: 1β (*Il-1b),* **E**: 18 (*Il-18)* and **F**: 6 *(Il-6)* and **G**: tumor necrosis factor ɑ (*Tnf-α)* in young and aged mice (n=7-8). Brain mRNA expression of **H**: *Nlpr3*, **I**: *Asc*, **J**: *Casp-1*, **K**: *Il-1b,* **L**: *Il-18*, **M**: *Il-6* and **N**: *Tnf-α* in young and aged mice (n=7-8). All data are mean ± S.E.M. *P<0.05 vs young, Student’s unpaired t-test.

Some inflammasome components (including *Nlrp3, Casp-1*; Fig. 5A-C)*,* and products of inflammasome activity (*Il-1β, Il-6*, but not *Il-18,* Fig. 5D-F)*,* as well as *Tnf*-*α* (Fig. 5G) were elevated in kidneys from aged mice. Aging did not change brain expression of *Nlrp3* (Fig. 5H) or *Il-18* (Fig. 5L) but increased expression of *Asc, Casp-1, Il-1β, Il-6* and *Tnf*-*α* (Fig. 5I, J, K, M, N).

**Figure:**
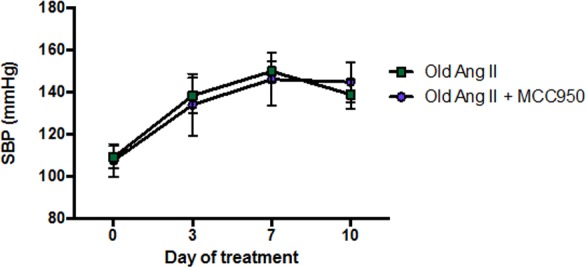


### Effect of an inhibitor of the NLRP3 inflammasome on Ang II-induced hypertension in old mice

Co-administration of Ang II and the selective NLRP3 inflammasome inhibitor, MCC950, to aged mice for 10 d had no effect on SBP compared with mice receiving vehicle and Ang II (Fig. 6).

## DISCUSSION

The major finding of this study is that, compared with young adult mice, aged mice exhibit a markedly augmented pressor response to infusion of a low, ‘slow-pressor’ dose of Ang II. This effect of aging is associated with increased vascular superoxide generation and larger vasoconstrictor responses to Ang II, increased expression of pro-inflammatory a higher ratio of AT1/AT2 receptor expression in blood vessels. However, despite evidence of chronic inflammation and increased expression and activity of the NLRP3 inflammasome complex in aged mice, the lack of effect of the inhibitor MCC950 on the pressor response to Ang II suggests that the NLRP3 inflammasome does not contribute to the augmented hypertensive response.

We found that whereas a slow-pressor dose of Ang II gradually increased blood pressure in young adult male mice, as has been reported [9], it caused a prompt and marked increase in blood pressure in aged male mice. The AT1R is the main target receptor of Ang II and it plays a key role in blood pressure regulation, vasoconstriction and oxidative stress [10–12], whereas the AT2R typically plays an opposing, protective role in such responses [13–15]. We confirmed that renal AT1R expression is increased in old age [3, 16] – a known effect of the actions of IL-1β and TNF-α [17] – both of which were also upregulated here. Moreover, there was a profound reduction in vascular AT2R expression during aging such that the ratio of AT1R/AT2R expression was markedly increased in mesenteric arteries, providing a plausible mechanism for the markedly augmented pressor and contractile responses to infusion of low-dose Ang II. Such a phenomenon is analogous to findings that male AT2R-deficient mice have higher baseline blood pressure and increased pressor responses to Ang II when compared to wild-type mice [18]. Indeed, a level of reduced AT2R signalling in old age may contribute to higher pressor responses to a high dose of Ang II [3].

The oxidative stress hypothesis of aging proposes that a progressive and irreversible build-up of oxidative stress mediated-damage causes aging and contributes to a decline in physiological function, increased morbidity and reduced life span [20]. While many studies have demonstrated that aging and reduced life span are correlated with elevated oxidative stress and damage, a direct cause and effect relationship has not been established [20]. We found that in both large capacitance (i.e. aorta) and smaller resistance-like (i.e. second-order mesenteric arteries) blood vessels, the reactive oxygen species (ROS)-generating capacity was augmented by >2-fold in aged mice. It has been reported that aortic superoxide levels are ~65% greater in aged mice compared to young mice [21]. Similarly, we found a trend for a ~60% higher basal level of ROS production in the isolated aorta of aged mice. Furthermore, the capacity of the vessels for NOX2-dependent ROS production was clearly shown to be augmented, when enzyme activity was driven by PdB. Thus, it is conceivable that increased vascular ROS production in vivo contributed to the exacerbated hypertension in aging. The greater efficacy of the NOX2 oxidase activator, PdB, to increase vascular superoxide levels in aged mice was not, however, correlated with any change in the mRNA expression of NOX2 (gp91phox) or other subunits of the NOX2 oxidase (p47phox, p67phox), although this does not necessarily reflect protein expression of NOX2 which has been reported to be increased in the aorta during aging [19].

Constrictor responses of the mesenteric arteries to Ang II were markedly and selectively greater in aged versus young adult mice. Although vascular expression of the *At1ar* was not higher in aged mice, there was markedly lower expression of the *At2r*, resulting in an increased ratio of vascular AT1R/AT2R expression with aging. Our finding that Ang II-induced vasoconstriction is selectively augmented with aging can be accounted for by the lower expression of AT2R. The opposing role of the AT2R is demonstrated in findings where overexpression of AT2R in vascular smooth muscle increases vasodilation and inhibition of AT2R augments Ang II-induced aortic constriction [22]. The enhanced Ang II-mediated vascular contraction was apparently not due to differing levels of vascular nitric oxide, prostacyclin or ROS production as acute application of L-NAME, indomethacin or tempol/catalase did not affect Ang II-induced vasoconstriction in either young or aged mice. Enhanced Ang II-induced vasoconstrictor responses in small arteries of the mesenteric bed would seem likely to have contributed to the augmented pressor response to Ang II in aged mice. Such an effect may be restricted to smaller arteries as contractile responses of carotid arteries to Ang II are reportedly similar in young and aged mice [23]. It is noteworthy that we do not suspect a role for the AT1bR in the augmented Ang II-induced responses observed here. Studies have shown that the AT1bR plays a much less important role in blood pressure control than the AT1aR [24]. Furthermore, we also examined AT1bR expression here and found no aging-related change in the kidneys, brain or blood vessels (data not shown).

Aging is characterized by persistent, low-grade inflammation, even in the absence of infection or apparent disease [25, 26]. We found evidence of inflammation in the kidneys and brains of aged mice. In particular, renal expression of components of the NLRP3 inflammasome, *Nlrp3* and *Caspase-1*, were upregulated. We recently showed that treatment of mice with MCC950, a selective NLRP3 inflammasome inhibitor [27], can reverse one kidney/DOCA/salt-induced hypertension in adult mice [7], implicating a role for inflammasomes in at least one form of hypertension. However, here we found that MCC950 did not affect blood pressure in aged mice when co-infused with Ang II, suggesting that NLRP3 inflammasome activity does not contribute to the increased pressure seen in this form of hypertension. In young adult mice, expression of *Nlrp3, Asc* and *Caspase-1* increased and inflammation developed after infusion of DOCA/salt [7], however, here we found that inflammation was already well-established in aged mice prior to challenge with a hypertensive stimulus. Perhaps, MCC950 is effective at lowering blood pressure in specific models of hypertension where inflammation is provoked by the hypertensive stimuli and not in animal models where chronic inflammation has developed before the onset of hypertension.

The prevalence of hypertension increases with age where more than 50% of the elderly have hypertension [1], and based on the findings of this study, one contributing factor could be increased AT1R and/or decreased AT2R expression in the elderly. AT1R antagonists have been reported to be more effective than some of the classical anti-hypertensive agents at improving clinical outcomes in the elderly [28], consistent with the concept that there is increased AT1R activity in the elderly.

In summary, we have found aged mice to have a markedly enhanced pressor response to Ang II which is associated with selectively augmented vasoconstrictor responses to Ang II, higher vascular ROS generation and an increased ratio of vascular AT1R/AT2R expression. There was also evidence of inflammation of renal and brain tissue, but an inhibitor of the NLRP3 inflammasome did not affect Ang II-induced hypertension in aged mice.

## MATERIALS AND METHODS

### Animals

This study was approved by the Monash University Animal Research Platform Animal Ethics Committee. Young adult (8-12 weeks old, n=33) and aged male C57Bl6J mice (23-31 months old, n=48) were obtained from Monash Animal Research Platform. Normal chow and drinking water were provided *ad libitum*.

### Drug administration and blood pressure measurement

Under anesthesia with isoflurane (2-4% inhaled with O_2_), an osmotic minipump (Alzet model 2004, ALZET, USA) was placed subcutaneously in the mid-scapular region to administer vehicle (0.9 % saline) or a slow-pressor dose of Ang II (0.28 mg/kg/d, Auspep, Australia) for 28 d. SBP was measured in conscious mice via tail cuff plethysmography using the MC4000 Multichannel system (Hatteras Instruments, USA). Prior to surgery, mice were trained for 1 d to acclimatize to the procedure, and blood pressure was then recorded on days 0, 3, 7, 10, 14, 17, 21, 24 and 28 of treatment. A cohort of aged mice (n=13) were implanted with two osmotic minipumps (Alzet model 2002) containing either Ang II (0.28 mg/kg/d) + vehicle (0.9 % saline) or Ang II (0.28 mg/kg/d) + MCC950 (10 mg/kg/d, a gift from Prof Matthew Cooper and Dr Avril Robertson) for 10 d. In these mice, SBP was measured on days 0, 3, 7 and 10 of treatment. Mice were euthanized by overdose of inhaled isoflurane (Baxter Healthcare, Australia).

### Gene expression

mRNA expression of various genes in the kidney, brain and mesenteric artery was determined using TaqMan® real-time PCR. Kidneys and brains were harvested and snap frozen in liquid nitrogen. Mesenteric arteries were dissected in RNAlater (Qiagen, USA) and either frozen or kept at 4°C in RNAlater. RNA was extracted from the kidneys and brains using RNeasy Mini kit (Qiagen, USA). Mesenteric arteries were homogenized in TRIzol (Life Technologies, USA), mixed with chloroform, and centrifuged at 13,000 rpm for 15 min at 4°C. The aqueous phase was collected and RNA was extracted using the RNeasy Micro kit (Qiagen, USA). RNA was quantified using the Nanodrop 1000D spectrophotometer (Thermo Scientific, USA) and converted to 1^st^ strand cDNA using High Capacity cDNA RT Kit (Applied Biosystems, USA). Commercially available primers (Applied Biosystems, USA) were used to measure mRNA expression of inflammatory markers, and a house-keeping gene, either β-actin or GAPDH, on a CFX96 Touch Real-Time PCR Detection machine (Bio-Rad, USA). Changes in gene expression were assessed using the comparative C_T_ method [29].

### Vascular function

Rings of second-order mesenteric arteries were isolated to measure contractile responses. Mesenteric arteries were mounted in a Mulvany-style small vessel myograph (Danish Myo Technology, Denmark) containing Krebs-bicarbonate buffer (in mmol/L: D-glucose 11.1, CaCl_2_ 2.5, NaCl 118, KCl 4.5, KH_2_PO_4_ 1.03, MgSO_4_ 0.45, NaHCO_3_ 25) and bubbled with carbogen (95% O_2_, 5% CO_2_) to measure isometric tension. Two arteries from each animal were mounted and their responses were averaged. After 20 min equilibration, arteries were stretched to a resting tension of ~2 mN, contracted with KPSS and then maximally contracted with U46619 (U-max, 300 nM, Cayman Chemical, USA). Cumulative concentration-response curves to Ang II, phenylephrine (Sigma Aldrich, USA) and U46619 were obtained with a 20 min washout period between each curve. In another set of experiments, the curves to Ang II, phenylephrine and U46619 were constructed in the absence and presence of L-nitro-arginine methyl ester (L-NAME, 300 μM, Sigma Aldrich, USA), indomethacin (3 μM, Sigma Aldrich, USA), tempol (1 mM, Sigma Aldrich, USA) or tempol (1 mM) + catalase (2500 U/ml, Sigma Aldrich, USA). Each of these drugs was pre-incubated for 30 min before each curve. Responses were expressed as a percentage of the U-max.

### Superoxide measurements

After euthanasia at d 28, L-012-enhanced chemiluminescence was used to measure superoxide levels in segments of thoracic aorta and mesenteric artery in the absence and presence of phorbol 12,13-dibutyrate (PdB, 10^−5^M, Sigma Aldrich, USA). In semi-darkness, aortic rings were placed in a white 96-well plate containing Krebs-HEPES buffer (in mmol/L: D-glucose 11.1, Na-Hepes 20, NaHCO_3_ 25, CaCl_2_ 2.50, NaCl 99.01, KCl 4.69, KH_2_PO_4_ 1.03, MgSO_4_ 1.20, pH 7.4) for measurement of background luminescence using a Hidex Chameleon single photon counter (30 × 1 min cycles, 3 s per well). L-012 (10^−4^M, Wako Pure Chemicals, Japan) was then added to each well and luminescence, reflecting basal superoxide production, was again measured for another 30 cycles. PdB was then added to each well to stimulate NADPH oxidase activity, and luminescence was measured for another 30 cycles. For mesenteric arteries, background counts were initially measured (30 × 1 min cycles, 3 s per well) in a white 96-well plate containing Krebs-HEPES buffer (composition as above) and L-012 with or without PdB. From each animal, one second-order mesenteric artery was harvested and cut in half by length. In semi-darkness, one half of the mesenteric artery segment was placed in a well containing Krebs-HEPES buffer and L-012 and the other half was placed in a well containing Krebs-HEPES buffer, L-012 and PdB. Measurements were made for another 30 cycles. The arteries were transferred onto foil on which they were oven-dried for 24 h at 37 ˚C in order to obtain dry tissue weights.

### Data presentation and statistical analyses

All data are expressed as mean ± s.e.m. Statistical analyses between groups were performed using Student’s unpaired t-test or a one- or two-way ANOVA followed by a Tukey’s or Bonferroni’s post-hoc test, as appropriate. P<0.05 was considered to be statistically significant. GraphPad Prism software version 6.0 was used to conduct all statistical analyses.

## SUPPLEMENTARY MATERIALS



## References

[1] van Rossum CT, van de Mheen H, Witteman JC, Hofman A, Mackenbach JP, Grobbee DE (2000). Prevalence, treatment, and control of hypertension by sociodemographic factors among the Dutch elderly. Hypertension.

[2] Dinh QN, Chrissobolis S, Diep H, Chan CT, Ferens D, Drummond GR, Sobey CG (2017). Advanced atherosclerosis is associated with inflammation, vascular dysfunction and oxidative stress, but not hypertension. Pharmacol Res.

[3] Mirabito KM, Hilliard LM, Head GA, Widdop RE, Denton KM (2014). Pressor responsiveness to angiotensin II in female mice is enhanced with age: role of the angiotensin type 2 receptor. Biol Sex Differ.

[4] Dinh QN, Drummond GR, Sobey CG, Chrissobolis S (2014). Roles of inflammation, oxidative stress, and vascular dysfunction in hypertension. BioMed Res Int.

[5] Chan CT, Sobey CG, Lieu M, Ferens D, Kett MM, Diep H, Kim HA, Krishnan SM, Lewis CV, Salimova E, Tipping P, Vinh A, Samuel CS (2015). Obligatory role for B cells in the development of Angiotensin II-dependent hypertension. Hypertension.

[6] Krishnan SM, Sobey CG, Latz E, Mansell A, Drummond GR (2014). IL-1β and IL-18: inflammatory markers or mediators of hypertension?. Br J Pharmacol.

[7] Krishnan SM, Dowling JK, Ling YH, Diep H, Chan CT, Ferens D, Kett MM, Pinar A, Samuel CS, Vinh A, Arumugam TV, Hewitson TD, Kemp-Harper BK (2016). Inflammasome activity is essential for one kidney/deoxycorticosterone acetate/salt-induced hypertension in mice. Br J Pharmacol.

[8] Song F, Ma Y, Bai XY, Chen X (2016). The expression changes of inflammasomes in the aging rat kidneys. J Gerontol A Biol Sci Med Sci.

[9] Kawada N, Imai E, Karber A, Welch WJ, Wilcox CS (2002). A mouse model of angiotensin II slow pressor response: role of oxidative stress. J Am Soc Nephrol.

[10] Zhou Y, Chen Y, Dirksen WP, Morris M, Periasamy M (2003). AT1b receptor predominantly mediates contractions in major mouse blood vessels. Circ Res.

[11] Crowley SD, Gurley SB, Coffman TM (2007). AT(1) receptors and control of blood pressure: the kidney and more. Trends Cardiovasc Med.

[12] Zhang H, Schmeisser A, Garlichs CD, Plötze K, Damme U, Mügge A, Daniel WG (1999). Angiotensin II-induced superoxide anion generation in human vascular endothelial cells: role of membrane-bound NADH-/NADPH-oxidases. Cardiovasc Res.

[13] Widdop RE, Jones ES, Hannan RE, Gaspari TA (2003). Angiotensin AT2 receptors: cardiovascular hope or hype?. Br J Pharmacol.

[14] Matrougui K, Loufrani L, Heymes C, Lévy BI, Henrion D (1999). Activation of AT(2) receptors by endogenous angiotensin II is involved in flow-induced dilation in rat resistance arteries. Hypertension.

[15] Savoia C, Tabet F, Yao G, Schiffrin EL, Touyz RM (2005). Negative regulation of RhoA/Rho kinase by angiotensin II type 2 receptor in vascular smooth muscle cells: role in angiotensin II-induced vasodilation in stroke-prone spontaneously hypertensive rats. J Hypertens.

[16] Kim JM, Uehara Y, Choi YJ, Ha YM, Ye BH, Yu BP, Chung HY (2011). Mechanism of attenuation of pro-inflammatory Ang II-induced NF-κB activation by genistein in the kidneys of male rats during aging. Biogerontology.

[17] Gurantz D, Cowling RT, Varki N, Frikovsky E, Moore CD, Greenberg BH (2005). IL-1beta and TNF-alpha upregulate angiotensin II type 1 (AT1) receptors on cardiac fibroblasts and are associated with increased AT1 density in the post-MI heart. J Mol Cell Cardiol.

[18] Ichiki T, Labosky PA, Shiota C, Okuyama S, Imagawa Y, Fogo A, Niimura F, Ichikawa I, Hogan BL, Inagami T (1995). Effects on blood pressure and exploratory behaviour of mice lacking angiotensin II type-2 receptor. Nature.

[19] Yoon HE, Kim EN, Kim MY, Lim JH, Jang IA, Ban TH, Shin SJ, Park CW, Chang YS, Choi BS (2016). Age-associated changes in the vascular renin-angiotensin system in mice. Oxid Med Cell Longev.

[20] Kregel KC, Zhang HJ (2007). An integrated view of oxidative stress in aging: basic mechanisms, functional effects, and pathological considerations. Am J Physiol Regul Integr Comp Physiol.

[21] Fleenor BS, Seals DR, Zigler ML, Sindler AL (2012). Superoxide-lowering therapy with TEMPOL reverses arterial dysfunction with aging in mice. Aging Cell.

[22] Tsutsumi Y, Matsubara H, Masaki H, Kurihara H, Murasawa S, Takai S, Miyazaki M, Nozawa Y, Ozono R, Nakagawa K, Miwa T, Kawada N, Mori Y (1999). Angiotensin II type 2 receptor overexpression activates the vascular kinin system and causes vasodilation. J Clin Invest.

[23] Meyer MR, Fredette NC, Barton M, Prossnitz ER (2014). Endothelin-1 but not angiotensin II contributes to functional aging in murine carotid arteries. Life Sci.

[24] Ito M, Oliverio MI, Mannon PJ, Best CF, Maeda N, Smithies O, Coffman TM (1995). Regulation of blood pressure by the type 1A angiotensin II receptor gene. Proc Natl Acad Sci USA.

[25] Franceschi C, Campisi J (2014). Chronic inflammation (inflammaging) and its potential contribution to age-associated diseases. J Gerontol A Biol Sci Med Sci.

[26] Cevenini E, Caruso C, Candore G, Capri M, Nuzzo D, Duro G, Rizzo C, Colonna-Romano G, Lio D, Di Carlo D, Palmas MG, Scurti M, Pini E (2010). Age-related inflammation: the contribution of different organs, tissues and systems. How to face it for therapeutic approaches. Curr Pharm Des.

[27] Coll RC, Robertson AA, Chae JJ, Higgins SC, Muñoz-Planillo R, Inserra MC, Vetter I, Dungan LS, Monks BG, Stutz A, Croker DE, Butler MS, Haneklaus M (2015). A small-molecule inhibitor of the NLRP3 inflammasome for the treatment of inflammatory diseases. Nat Med.

[28] Tadevosyan A, Maclaughlin EJ, Karamyan VT (2011). Angiotensin II type 1 receptor antagonists in the treatment of hypertension in elderly patients: focus on patient outcomes. Patient Relat Outcome Meas.

[29] Schmittgen TD, Livak KJ (2008). Analyzing real-time PCR data by the comparative C(T) method. Nat Protoc.

