# Supplementary material for: Pressor response to angiotensin II is enhanced in aged mice and associated with inflammation, vasoconstriction and oxidative stress
Source: Aging (Albany NY). 2017 Jun 28;9(6):1595–605. doi: 10.18632/aging.101255 (PMC5509458; doi:10.18632/aging.101255)
Supplement: Supplementary file 1 [file aging-09-1595-s001.pdf]

SUPPLEMENTARY MATERIALS

Supplemental Figures

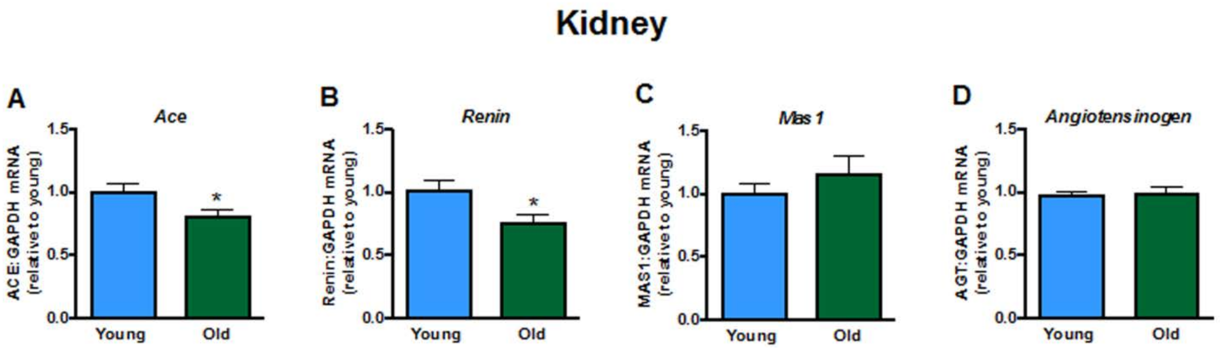

Supplementary Figure 1. Renal mRNA expression of **A:** angiotensin converting enzyme (*Ace*), **B:** *Renin,* **C:** mas receptor 1 *(Mas1)* and **D:** *Angiotensinogen.* All data are mean ± S.E.M. *P<0.05 vs young, Student's unpaired t-test.
